# The Gastroscope

**Published:** 1885-04

**Authors:** 


### The Gastroscope.

The *Med. Press and Circular* tells us that Mr. J. Leiter, of Vienna, has constructed a singular modification of the microscope, to which the name of gastroscope has been given. Its use is for exploring the interior of the stomach. It consists of a metal tube, 65 cm. long, and 15 mm. thick, bent at an angle of 150° about one fourth of its length from the lowest end. At the lower extremity is contained an incandescent electric lamp for illumination of the interior of the stomach and an objective, at the back of which is a prism to reflect the pencil along the length of the tube; at the bend it is again reflected by another prism to the eye-piece. Provision is made for a circulation of cold water to prevent the lower end of the tube becoming inconveniently hot.—*Med. and Surg. Rep.*

Carbuncles—Sponge-Grafting.—Surgeon-Major T. Cody (*Transactions, Medical and Physical Society of Bombay*, No. 11, new series) has had occasion to use this method in a case of carbuncle where, after the slough had detached itself, an excavation was left two and a half inches long by two broad, and varying in depth from a quarter of an inch in parts to an inch in others. The carbolized sponge was fitted almost exactly in the parts. To retain it in close apposition three silver-wire sutures were applied. In four days' time the sponge was firmly imbedded or grafted on the surface, so the sutures were removed. Repeated carbolized washings and dressings were employed, with removal by the scissors of such parts of the sponge as projected from time to time, until in six weeks' time the patient left the hospital with the surface completely healed over, and without the slightest puckering or depression to be seen in the cicatrix.

